# Polypyrrole Chains Decorated on CoS Spheres: A Core-Shell Like Heterostructure for High-Performance Microwave Absorption

**DOI:** 10.3390/nano10010166

**Published:** 2020-01-17

**Authors:** Hui Liu, Guangzhen Cui, Ling Li, Zhi Zhang, Xuliang Lv, Xinxin Wang

**Affiliations:** 1 Graduate School, The Army Engineering University of PLA, Nanjing 210007, China; liuhh1005@163.com (H.L.); cgzovezy@163.com (G.C.); zhangnjjn@163.com (Z.Z.); 18260093995@163.com (X.W.); 2 Engineering College of Field Engineering, The Army Engineering University of PLA, Nanjing 210007, China; xllu1957@126.com

**Keywords:** microwave absorption, CoS, PPy, core-shell structure

## Abstract

Cobalt sulfide composites have exhibited great potential in terms of microwave absorption, owing to their low price, relatively high capacitance, and excellent electrocatalytic activity. Thus, a novel core-shell like structure comprising cobalt sulfide@polypyrrole (CoS@PPy) composite was synthesized by a facile solvothermal synthesis method and in situ polymerization. When coated by the heterostructure polypyrrole aerogel, CoS@PPy composite exhibited excellent microwave absorption properties with an optimal reflection loss (R_L_) of −41.8 dB at 6.96 GHz. Furthermore, the absorption bandwidth (R_L_ < −10 dB) of 5.4 GHz could be reached at a coating thickness of 2.05 mm, probably attributing to the synergistic effect of good impedance matching, interfacial polarization, dipole polarization, and conductivity loss. Moreover, this work proposed a loss mechanism mode which probably occurred in the CoS@PPy composites. It was demonstrated that the CoS@PPy composite is a promising material in the field of electromagnetic wave absorption.

## 1. Introduction

With the wide range of applications of modern electronics, portable electronics and wireless technology play a dominant role in daily life [1,2]. However, the environment, human immune health, information security, and device performance are affected by increasingly serious electromagnetic interference pollution. Accordingly, considerable attention is focused on the application of electromagnetic absorption (EA) materials [3]. Good EA materials should be lightweight, have a low filler loading ratio, a wide frequency, and strong absorption [4,5,6,7]. In the last few years, traditional absorbing materials, such as ferrites [8,9,10], carbon fiber, reduced graphite oxide (RGO) [11], and conductive polymers [12], have been widely explored. However, their high density, corrodibility, high electric conductivity, and narrow absorption band restrict their application. Thus, the development of a kind of absorber with an excellent attenuation ability and impedance matching remains the focus of the search for future absorbing materials.

More recently, the microwave absorbing capacity of cobalt sulfide (CoS) nanocomposites has attracted great attention. As a kind of semiconductor transition metal sulfide, CoS offers good performance in energy storage systems [13], optoelectronic devices [14], lithium ion batteries [15], and catalysis [16] due to its low price, relatively high capacitance, excellent electrocatalytic activity, and conductivity. Significantly, CoS with various sizes and morphologies, including nanoparticles [17], hollow microspheres [18], 3D flowerlike hierarchitectures [19], nanoboxes [20], and wormlike microtubes [21], have been successfully synthesized. For instance, Huang et al. fabricated flowerlike CoS hollow spheres with an effective absorption bandwidth of 4.6 GHz, with the maximum reflection loss (R_L_) of the materials reaching −43.6 dB when the thickness was 2.0 mm. Hierarchical wormlike cobalt sulfide microtubes were synthesized by Liu et al., who demonstrated the potential of template-assisted synthesis [21]. Recently, a lightweight Co-C/Co_9_S_8_ composite with a tunable cavity possessed extremely promising microwave absorption ability at a low frequency [22]. However, broadening the effective absorption bandwidth and maintaining good impedance matching remain important challenges in the design of CoS composite absorbing materials.

Thus far, increasing efforts have been made in relation to the synthesis of conduction polymer composites, such as those made of polyaniline (PANI) [23], polyvinylidene fluoride (PVDF) [24], polypyrrole(PPy) [25], and poly(3,4-ethylenedioxythiophene) (PEDOT) [26]. Wu et al. prepared a CoS/PVDF composite by a simple blending method and a maximum RL was −43 dB with a low loading of only 5.0 wt % [27]. The permittivity and permeability are important indices to evaluate microwave absorption materials. Owing to its low cost, light weight, and high electrical conductivity, polypyrrole (PPy) has attracted significant attention as a potential absorbing material [28]. When external electromagnetic fields exist, polyrrole can offer a synergistic effect of a conjugated bond and electric dipole in the composite materials. As a result of the poor impedance matching, high conductivity, and limited dielectric loss of pure polypyrrole material, its composites have been widely investigated to optimize the absorption performance, such as s-PPy@RGO [29], PPy@SiC [30], Co@PPy [31], CuS@PPy [32], and Ti3C2Tx @PPy [33]. In fact, various materials with different loss properties can be combined to synthesize excellent absorbers that exhibit a remarkable attenuation capacity and impedance matching. Herein, we propose a scheme in which chain-shaped polypyrrole can grow on the surface of a cobalt sulfide matrix. In this way, a rich conductive network structure can be constructed to improve the attenuation capacity of electromagnetic waves.

**Figure 1:**
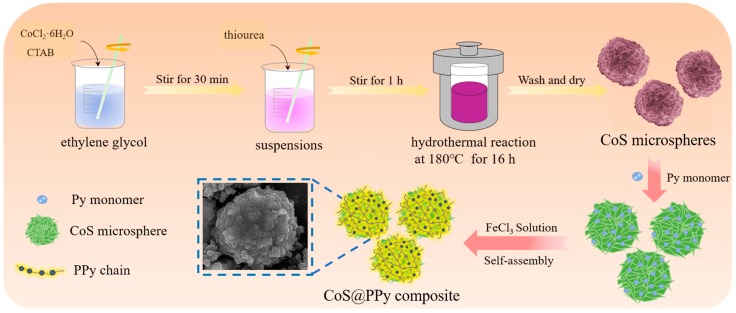
Schematic diagram of preparation process of CoS@PPy composites.

As far as we know, there are no reports about the exploration of cobalt sulfide@polypyrrole (CoS@PPy) for electromagnetic wave absorption. In this work, the core-shell-structured CoS@PPy microspheres were prepared via solvothermal and self-assembled polymerization (Figure 1). As confirmed by comprehensive characterization, CoS microspheres were successfully coated with polypyrrole with an amorphous structure. The maximum reflection loss of CoS@PPy composites was −41.8 dB at 6.96 GHz and the effective absorbing bandwidth (<−10 dB) was 5.4 GHz with a thickness of only 2.05 mm. The excellent EA ability of CoS@PPy composites can be ascribed to excellent impedance matching, intensified electric dipoles, and interfacial polarizations. There are reasons to believe that the CoS@PPy composites would be useful in military and civil fields as a promising microwave absorber.

## 2. Materials and Methods

### 2.1. Materials

Cobalt chloride hexahydrate (CoCl_2_·6H_2_O), cetyltrimethyl ammonium bromide (CTAB), and pyrrole (Py) monomer were obtained from Aladdin. Ferric chloride hexahydrate (FeCl_3_·6H_2_O), thiourea (CN_2_H_4_S), ethylene glycol (EG), and absolute ethanol (CH_3_CH_2_OH) were offered by Sinopharm Chemical Reagent Factory. All the reagents were of analytical grade and were used without further treatment.

### 2.2. Synthesis of CoS Microspheres

CoS microspheres were synthesized using the hydrothermal method [18]. In a typical experiment, 0.004 mol of CoCl_2_·6H_2_O was first dispersed in 65 mL EG. Subsequently, 0.004 mol of CTAB was dispersed in EG (65 mL), and added into the above system under sonication for 30 min. Then, 0.01 mol of thiourea was introduced into the suspensions with vigorous stirring for 1 h. Afterwards, the solution was sealed into a 200 mL Teflon-lined autoclave and maintained for 16 h at 180 °C. Finally, the cooled products were washed several times using absolute ethanol and deionized water, and dried at 60 °C for 12 h.

### 2.3. Synthesis of CoS@PPy Microspheres

The prepared CoS microspheres (80 mg) were dispersed in 25 mL of anhydrous ethanol and sonicated for 30 min. Whereafter, Py monomer (82.5 mg) was added to the CoS dispersion to form mixture A. Then, 775 mg of FeCl_3_·6H_2_O dissolved in 25 mL of deionized water was mixed with the above solution dropwise under rapid stirring. After in situ polymerization for 24 h, the precipitate was washed several times with alcohol and water and then dried at 50 °C for 12 h under vacuum. Figure 1 shows a schematic diagram of the preparation process of CoS@PPy composites.

### 2.4. Material Measurement

The chemical compositions of the samples were examined using an X-ray diffractometer (XRD, D/MAX-UltimaIV, Tokyo, Japan). We used field emission scanning electron microscope (FE-SEM, Quanta F400) and transmission electron microscopy (TEM, Hitachi HT7800, Tokyo, Japan) to investigate the surface morphology of the materials. Fourier transform infrared (FTIR) was performed by Nicolet iS10 FITR instrument (Thermo Fisher Scientific, MA, USA). X-ray photoelectron spectroscopy (XPS, VG ESCALab 220i-XL, East Sussex, UK) was used to investigate the element states of the surfaces.

The electromagnetic parameters were calculated at room temperature using an Agilent E8362B PNA vector network analyzer via the transmission/reflection line method in the frequency range 2–18 GHz. The samples were ground into powder and mixed with paraffin wax in different filling ratios. Then, a special mold was used to artificially press the sample–paraffin max composites into a coaxial ring with an inner diameter of 3.04 mm, an outer diameter of 7.00 mm, and a thickness of 3.00 mm. According to the transmission line technique, the reflection loss (R_L_) reflectivity of the samples was calculated by following formula [34]:

(1) $$Z_{in}=Z_{0}\sqrt{\frac{\mu_{r}}{\varepsilon_{r}}}\tan h\;[j\frac{2\pi fd}{c}\sqrt{\mu_{r}\varepsilon_{r}}],$$

(2) $$R_{L}=20\log\left|\frac{Z_{in}-Z_{0}}{Z_{in}+Z_{0}}\right|,$$

where *Z*_0_ represents the free space impedance; *Z_in_* represents the input characteristic impedance; *ε_r_* and *μ_r_* are the complex relative permittivity and permeability of the absorber, respectively; *c* is the light velocity in a vacuum; *f* is the frequency of the electromagnetic wave; and *d* denotes the layer thickness.

## 3. Results and Discussion

### 3.1. Characterization of Samples

**Figure 2:**
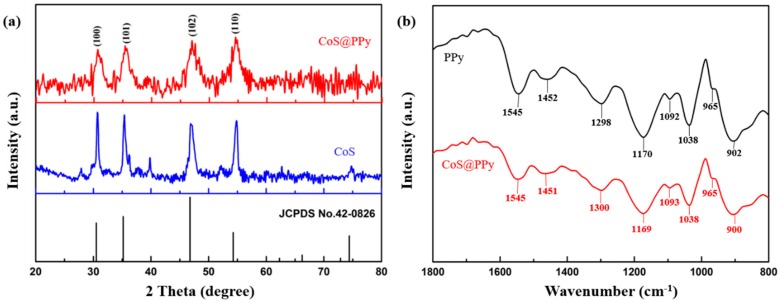
(**a**) XRD results of CoS and CoS@PPy and (**b**) FTIR spectra of PPy and CoS@PPy.

The crystal structure of CoS and CoS@PPy composites were confirmed by XRD and the results are shown in Figure 2a. The diffraction peaks at 31.2°, 35.3°, 47.1°, and 54.9° were consistent with the standard XRD data for the (100), (101), (102), and (110) planes of hexagonal CoS (JCPDS card No. 42-0826). Notably, the intensities of all the diffraction peaks for CoS@PPy composites were weaker than those of the CoS, which might be due to the amorphous structure of PPy affecting the crystallinity of CoS. The FTIR spectra of PPy and CoS@PPy are shown in Figure 2b. It can be seen that the curves look similar and the peaks change slightly, which might be due to the interaction between the polypyrrole rings and CoS [29]. The bands at 1545 and 1451 cm^−1^ can be ascribed to the symmetric and antisymmetric stretching vibrations of PPy rings. In addition, peaks at 1300 and 1038 cm^−1^ were attributed to the C–N stretching and C–H deformation vibrations, respectively [35]. Peaks at 1169 and 900 cm^−1^ indicate the doping state of the aerogel. The peaks at 1093 and 965 cm^−1^ correspond to the protonation in the PPy chain (NH_2_^+^) and the C–C out-of-plane ring deformation vibration, respectively [36].

**Figure 3:**
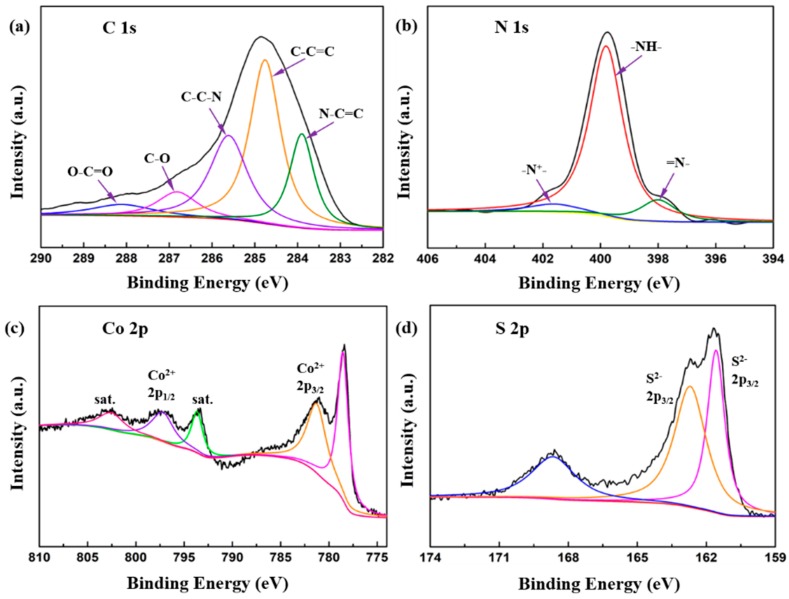
XPS spectra of CoS@PPy: (**a**) C 1s; (**b**) N 1s; (**c**) Co 2p; (**d**) S 2p.

The surface elemental composition was investigated by XPS and is presented in Figure 3. The high-resolution spectrum of the C 1s could be divided into five different peaks at 283.76, 284.65, 285.64, 286.95, and 288.29 eV, corresponding to N–C=C, C–C=C, C–C−N, C–O, and O–C=O, respectively. The broad band of N 1s is shown in Figure 3b. The typical peak at 398 eV, which corresponds to the quinoid imine (–N=) and the benzenoid amine (–NH–), was observed at 399.98 eV. Meanwhile, another peak located at 401.5 eV is due to the cationic nitrogen atoms (–N^+^–) [37]. In the Co 2p spectra (Figure 3c), the peak at 778.5 eV was ascribed to sulfide Co–S [21]. The peak at 781.2 eV corresponds to the Co 2p_3/2_, and the peak at 797.2 eV is characteristic of Co 2p_1/2_. Additionally, two shake-up satellites peaks at 793.6 and 802.5 eV are related to Co^2+^ present in the CoS@PPy sample [38,39]. Figure 3d represents a high-resolution spectrum of S 2p, with peaks at 161.5 and 162.7 eV indicating that sulfur exists as S^2−^ in the sample. A peak could also be observed for O impurities as the sample surface was in contact with the air during processing [40]. On the basis of the above analyses, we can confirm that the CoS@PPy composites were successful synthesized.

**Figure 4:**
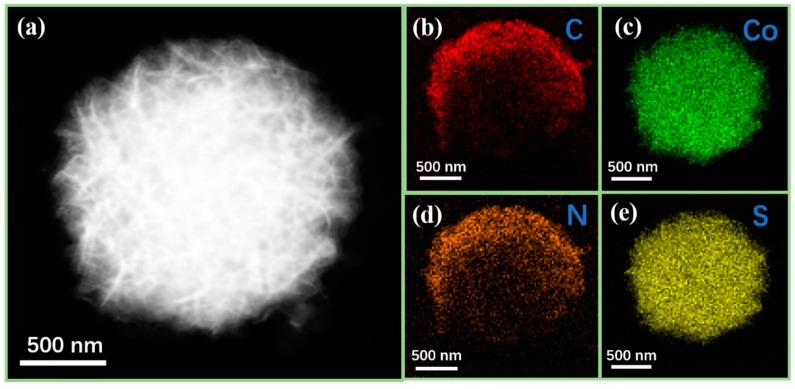
STEM of CoS@PPy (**a**) and elemental mapping images of (**b**) C, (**c**) Co, (**d**) N, (**e**) S.

The element composition of CoS@PPy composites are presented in Figure 4. It is obvious that Co elements (Figure 4c) and S elements (Figure 4e) were equally distributed in the mixture. By contrast, the distribution range of C and N elements were significantly larger than that of Co and S elements, as shown in Figure 4b,d. It confirms that CoS microspheres were successfully combined with polypyrrole, which might be help to improve the absorption properties of the polymer.

**Figure 5:**
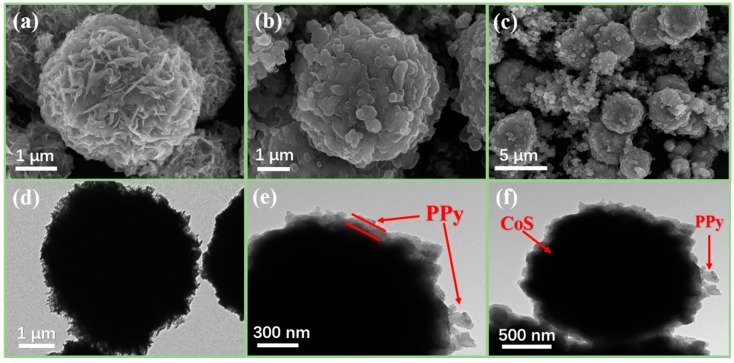
SEM images of CoS (**a**); CoS@PPy (**b**,**c**); TEM images of CoS (**d**); and CoS@PPy (**e**,**f**).

The morphological features and nanostructures of CoS microspheres and CoS@PPy composites were observed using SEM and TEM. As revealed in Figure 5a, the average diameter of hierarchical flowerlike CoS microspheres was about 4 μm, and they were assembled from numerous thin interleaving nanoflakes with a thickness of about 35 nm. As illustrated in Figure 5b, the samples still maintained a micron spherical structure after the gentle polymerization process. With the CoS microsphere as a matrix, PPy was oxidized and polymerized on the surface of the CoS microsphere, resulting in a core-shell structure. From Figure 5c, it can be seen that the redundant polypyrrole was interconnected and clustered into a banded structure scattered around the microspheres. The microstructure and morphology of the sample were further revealed by the TEM images. On the basis of Figure 5d, regular flowerlike hierarchitectures can be confirmed. Figure 5e,f display the TEM image of CoS@PPy composites, which confirms that the CoS microspheres were encased in the polypyrrole, with a thickness of around 20–90 nm.

### 3.2. Electromagnetic Parameters and Absorption Property

**Figure 6:**
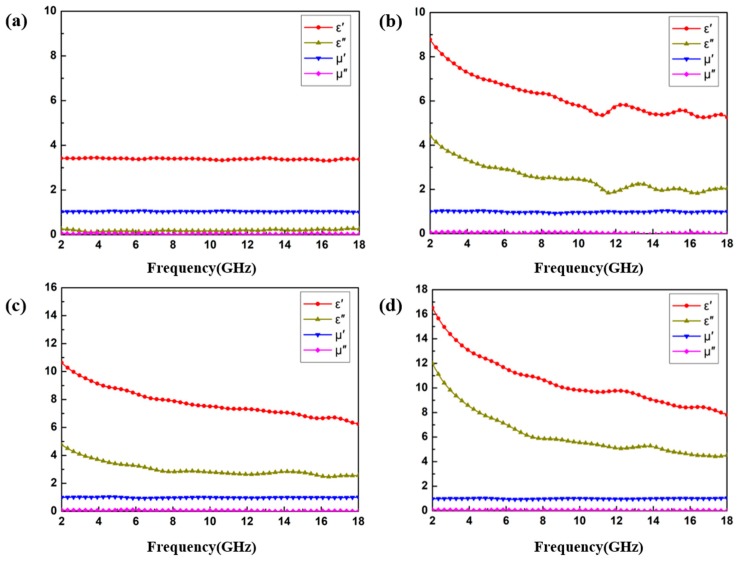
(**a**) The real and imaginary parts of the complex permittivity and complex permeability of CoS (15 wt %); The real and imaginary parts of the complex permittivity and complex permeability of CoS@PPy with the filler loading of (**b**) 15 wt %, (**c**) 20 wt %, (**d**) 25 wt %.

The frequency dependence of the real and imaginary part of the complex permittivity, and the complex permeability corresponding to the CoS and CoS@PPy samples with different filler loadings is shown in Figure 6. It is known that the real part of permittivity *ε*′ is responsible for energy storage, and the imaginary part of permittivity *ε*″ is associated with energy dissipation. Noticeably, the real and imaginary parts of *μ_r_* were close to one and zero, indicating the negligible contribution of magnetic loss [41]. As illustrated in Figure 6a, the values of *ε*′ remained around 3.5 with no significant fluctuation at the frequency range 2–18 GHz, while the values of *ε*″ were close to zero. Hence, the CoS sample (15 wt %) had almost no dielectric loss capacity. Compared to the CoS (15 wt %), the *ε*′ and *ε*″ values of CoS@PPy composites were obviously improved according to Figure 6b. In addition, as described in Figure 6b–d, the values of *ε*′ and *ε*″ increased with the rising mass fraction (15 wt % to 25 wt %), which could be due to the improved electrical conductivity of the materials and dipolar polarization. As we all know, polypyrrole has excellent electrical conductivity, which is affected by carrier mobility. After the cobalt sulfide was coated with polypyrrole, it can be speculated that a conductive network path was formed in the composites as the doping ratio increased, which could be more conducive to the electron transport between the CoS@PPy microspheres [29,42]. In an alternating electromagnetic field, the polarization capability of samples can be characterized by the values of *ε*′ [43]. More electron migration leads to more electron polarization, which is the reason that the values of *ε*′ increase with the rising mass fraction.

The polarization process is mainly categorized into the following types: electronic polarization, ionic polarization, dipole polarization, and interfacial polarization [44]. As previously reported [33], ionic polarization and electronic polarization occur at 10^3^–10^6^ GHz. Therefore, dipole orientation polarization and interfacial polarization play a major role in the polarization process of the materials. According to the frequency dispersive effect, induced charges lose their ability to react in time to the alternating EM field and the polarizability is unsustainable [45]; thus, the values of *ε*′ decreased with the increasing frequency, as shown in Figure 6. The hysteresis phenomenon, in which the rotation speed of the dipole lags behind the changing speed of the electromagnetic field, is called polarization relaxation [46], and is an important source of dielectric loss. The imaginary part of complex permittivity *ε*″ is associated with energy dissipation, which can be subject to the synergistic effect of polarization relaxation and conductivity loss [47]. Figure 6 shows that the values of *ε*″ increase with the rising mass fraction, indicating that the dielectric loss capability of CoS@PPy composites improved.

In order to better explain the polarization loss mechanism in the CoS@PPy composites, the Cole-Cole semicircle model was used. According to the Debye theory, the complex dielectric constant *ε_r_* is associated with the conductivity and the dielectric polarization. The *ε*′ and *ε*″ can be described by the following equation:

(3) $$\varepsilon^{\prime }=\varepsilon_{\infty }+\frac{\varepsilon_{s}-\varepsilon_{\infty }}{1+\omega^{2}\tau^{2}},$$

(4) $$\varepsilon^{\prime \prime }=\frac{\omega \tau \left(\varepsilon_{s}-\varepsilon_{0}\right)}{1+\omega^{2}\tau^{2}},$$

where $\varepsilon_{\infty }$ stands for the relative dielectric constant; $\varepsilon_{s}$ is the static permittivity; ω represents the electric field oscillation frequency; and τ stands for the polarization relaxation time. From Equations (3) and (4), the relationship between *ε*′ and *ε*″ may be expressed as

(5) $${\left(\varepsilon^{\prime }-\frac{\varepsilon_{s}+\varepsilon_{\infty }}{2}\right)}^{2}+{\left(\varepsilon^{\prime \prime }\right)}^{2}={\left(\frac{\varepsilon_{s}-\varepsilon_{\infty }}{2}\right)}^{2}.$$

**Figure 7:**
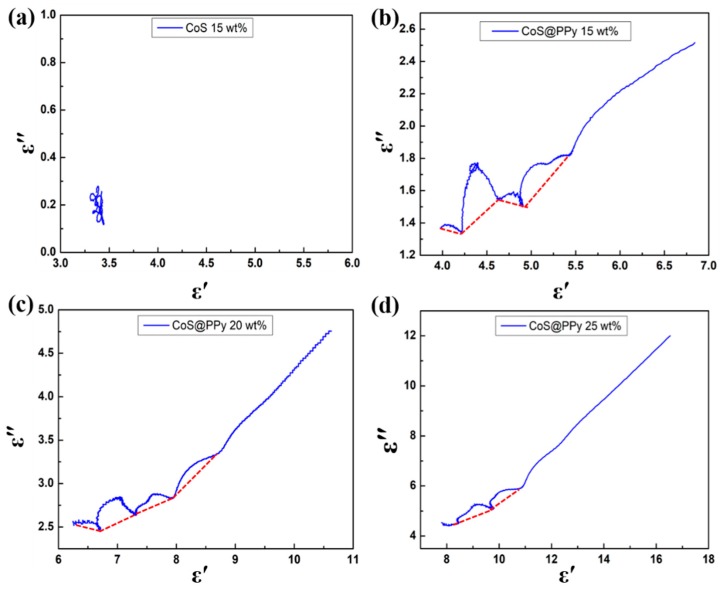
(**a**) The Cole-Cole plots of CoS (15 wt %); the Cole-Cole plots of CoS@PPy with the filler loading of (**b**) 15 wt %, (**c**) 20 wt %, (**d**) 25 wt %.

Figure 7 shows that the plot of *ε*′ versus *ε*″ can be seen as a single semicircle and each semicircle corresponds to a Debye relaxation process [48]. In Figure 7a, the semicircles of CoS (15 wt %) were hardly to be found, which indicated that the polarization loss could be neglected. From Figure 7b, CoS@PPy (15 wt %) exhibited four Cole-Cole semicircles, indicating that there were multirelaxation polarization processes occurring in the materials. As shown in Figure 7c, there were four semicircles in the CoS@PPy composites that might be attributed to the enhanced interfacial polarization between CoS and PPy [49]. Moreover, the number of semicircles in Figure 7d were significantly less than that in Figure 7b,c, probably because the effect of polarization loss was gradually hidden by the conductivity loss as the doping amount increased [11,29]. After explaining the polarization process, the absorption mechanism of the CoS@PPy composites still needs to be explored by dielectric loss and impedance matching.

**Figure 8:**
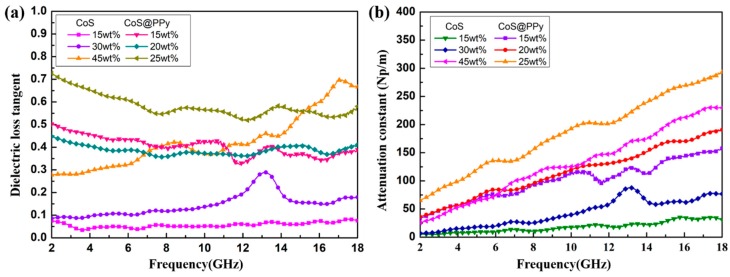
Dielectric loss tangent of CoS and CoS@PPy with different filler loadings (**a**); the attenuation constant of CoS and CoS@PPy with different filler loadings (**b**).

Electromagnetic wave loss and impedance matching properties are influenced by *ε_r_* and *μ_r_*. Dielectric loss played a leading part in the process of CoS@PPy composites absorbing waves. To further explore the absorption mechanism, the dielectric loss characteristics of composites were evaluated by the dielectric loss tangent ($\tan\delta_{\varepsilon }=\varepsilon^{\prime \prime }/\varepsilon^{\prime }$). Figure 8a shows dielectric loss tangent of CoS and CoS@PPy with different filler loadings. It can be seen that when the doping ratio increases from 15 wt % to 45 wt %, the $\tan\delta_{\varepsilon }$ of CoS improves obviously. By contrast, the dielectric loss capabilities of CoS@PPy composites were better, with $\tan\delta_{\varepsilon }$ in the range 0.5–0.75 (25 wt %). As seen in Figure 8a, $\tan\delta_{\varepsilon }$ showed relatively large fluctuations over the 6.0–18.0 GHz range, and the peaks near 10, 13, and 15 GHz could be suggested to originate from Debye relaxation. In addition to dipole orientation polarization, another contributor to dielectric losses is the interfacial polarization effect, also known as the Maxwell–Wagner–Sillars (MWS) effect [50,51], which usually happens in heterogeneous structures. In the external electric field, as a result of the differences in conductivity and dielectric properties, the charges will accumulate at the boundary of the two materials. The CoS@PPy materials were typical core-shell structures, and the charge distribution difference at the boundary would result in the interfacial polarization [52]. The concentrated motion of the interface dipole increases the response to the incident electromagnetic field and attenuates more electromagnetic waves.

Furthermore, the attenuation constant (*α*) of the absorbers is also an important parameter to evaluate the attenuation properties, which can be expressed by [53,54]

(6) $$\alpha =\frac{\sqrt{2}\pi f}{c}\times \sqrt{\left(\mu^{\prime \prime }\varepsilon^{\prime \prime }-\mu^{\prime }\varepsilon^{\prime }\right)+\sqrt{{\left(\mu^{\prime \prime }\varepsilon^{\prime \prime }-\mu^{\prime }\varepsilon^{\prime }\right)}^{2}+{\left(\mu^{\prime }\varepsilon^{\prime \prime }+\mu^{\prime \prime }\varepsilon^{\prime }\right)}^{2}}}.$$

**Figure 9:**
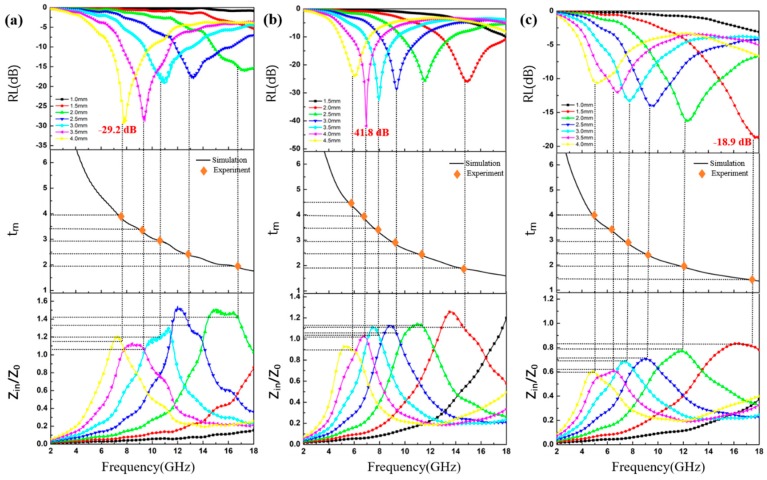
The reflection loss curves, the absorber thickness *t_m_*, and impedance matching curves obtained for various thicknesses for the CoS@PPy composites containing (**a**) 15 wt %, (**b**) 20 wt %, (**c**) 25 wt %.

As demonstrated in Figure 8b, as the frequency increased, all values of attenuation constants (*α*) increased with some fluctuations, especially at 10–18 GHz. Moreover, it was obvious that the value of attenuation constants also increased as the loading increased. When the doping ratio was 25 wt %, the attenuation constant was the highest. It can be seen that the attenuation capabilities of the CoS microspheres were relatively weaker than those of core-shell-structured CoS@PPy composites in the case of the same doping ratio. Combined with the Figure 9b, good impedance matching with a high capability of microwave attenuation suggests the superior absorption performance of CoS@PPy composites (20 wt %).

The variations in the calculated R_L_ curves of the CoS@PPy composites at different thicknesses and the comparison with the calculated matching thickness are presented in Figure 9. As the thickness increased, the reflection loss peaks moved toward the lower frequencies. Generally, absorbers with excellent properties can absorb more than 90% of microwaves when reflection loss (R_L_) is below −10 dB. From Figure 9a, the minimum R_L_ of CoS@PPy was −29.2 dB at 7.76 GHz, the corresponding coating thickness was 4.0 mm, and the effective absorbing bandwidth was 3.08 GHz when the doping ratio was only 15 wt %. As seen in Figure 9b, a doping ratio of 20 wt % yielded a reflection loss value of −41.8 dB at 6.96 GHz, which can meet the requirement of enhanced absorbing capacity. As seen in Figure 9c, as the doping ratio reached as high as 25 wt %, the maximum RL value of CoS@PPy was −18.9 dB at 17.9 GHz with the corresponding thickness of 1.5 mm, and the broad absorbing bandwidth (R_L_ < −10 dB) was only 3.12 GHz. This is because an excessively high permittivity can lead to an unsatisfactory impedance matching with a strong reflection character. It can be observed that the absorbing bandwidth of the reflection loss values less than −10 dB reached 5.12 GHz (from 12.88 to 18.0 GHz) when the corresponding coating thickness of the CoS@PPy composites (20 wt %) was only 2.0 mm. This means that CoS@PPy composites perform well, not only in terms of absorption depth, but also in absorption bandwidth, which makes CoS@PPy composites stand out among absorbing materials.

Moreover, simulation curves of the absorber based on the quarter-wavelength cancellation are shown in Figure 9, which can be expressed as follows [55]:

(7) $$t_{m}=\frac{n\lambda }{4}=nc/4f_{m}\sqrt{\left|\mu_{r}\right|\left|\varepsilon_{r}\right|}.$$

Here, $t_{m}$ is the absorber thickness and $f_{m}\;$ is the absorption attenuation frequency. The orange diamonds, located exactly on the 1/4 curve, exhibited matching thicknesses corresponding to the peak frequencies. Therefore, the effect of the R_L_ values on the thickness and frequency can be well illustrated by the quarter wavelength (*λ*/4) principle, suggesting that wave absorption frequency range can be regulated by reasonably calculating the thickness. As shown in Figure 9b, when the thickness was 4.0 mm, the relevant impedance matching ratio *Z* ($\left|Z_{in}/Z_{0}\right|$) was close to 1.0, confirming that excellent impedance matching is of great benefit in improving the absorption properties.

**Figure 10:**
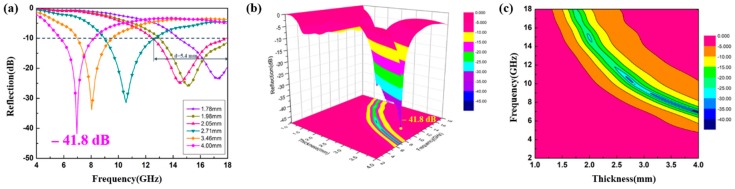
The calculated reflection loss of CoS@PPy composites at the loading of 20 wt % (**a**) and corresponding three-dimensional presentation (**b**) and its contour map (**c**).

In order to explore the influence of the thickness on the absorption performance of the core-shell-structured CoS@PPy composites more intuitively, the partial reflection loss diagram of the sample with a doping ratio of 20 wt % is shown in Figure 10a. Meanwhile, the three-dimensional presentations of R_L_ against the frequency and thickness and its contour map are illustrated in Figure 10b,c. The maximum R_L_ value of CoS@PPy was −41.8 dB at 6.96 GHz for 4.00 mm thickness, which may be due to the synergy effect of impedance matching and dielectric loss. Moreover, when the thickness was only 2.05 mm, the absorbing bandwidth below −10 dB was 5.4 GHz (12.48–17.88 GHz), almost covering the entire Ku band (12.75–18.00 GHz). From the results, it can be observed that with the increase in material thickness, the maximum R_L_ peaks moved towards the low frequency region. The above phenomenon can be proved by the formula $f=c/2\pi \mu_{r}{}^{\prime \prime }d$, in which the material thickness is inversely proportional to the corresponding frequency [56]. Concisely, the CoS@PPy composites (20 wt %) meet the requirements of good absorbing materials due to their light-weight nature, wide absorption bandwidth, and strong absorbing capacity.

**Figure 11:**
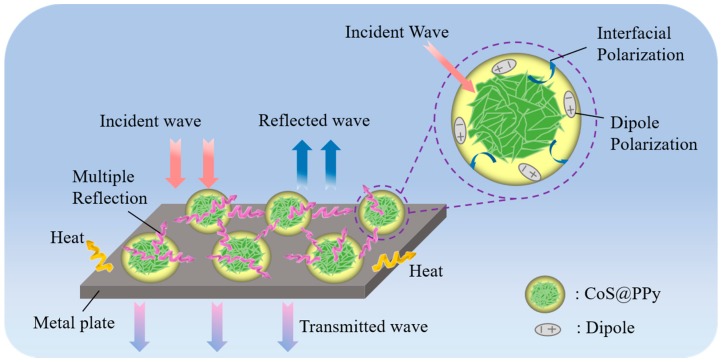
The absorption mechanism diagram of CoS@PPy composites.

As a result, it can be speculated that the microwave absorption mechanisms of CoS@PPy composites may include impedance matching, conductivity loss, and polarization loss. As shown in Figure 11, we propose a loss mechanism model that probably occurred in the CoS@PPy composites, which can be explained by the following aspects. Firstly, owing to excellent impedance matching, most of the incident electromagnetic waves went inside the material, with only a small fraction reflecting back. The incoming microwaves were refracted and scattered multiple times between the CoS@PPy microspheres and eventually dissipated as heat [57]. Secondly, it is well known that PPy has high conductivity. Under the applied electric field, electrons migrated on the surface of the CoS@PPy composites and constituted electric current. The formation of a conductive network could play a great role in the enhancement of conductivity loss. In addition, the introduction of polypyrrole might bring with it greater collective directional movement of carriers and enhance the dipole orientation polarization. Hence, the polarization relaxation loss would increase, and electromagnetic energy would be more easily converted into heat. Finally, because of the differences in conductivity, the electric fields on the interface were unevenly distributed. Free electrons accumulated at the interface between the CoS and PPy. According to the Debye theory, it can be concluded that the special core-shell structure of CoS@PPy probably induces interfacial polarization, which might improve the absorption ability. Admittedly, there is a need for more research to prove the possible absorption mechanisms.

## 4. Conclusions

In conclusion, hierarchal core-shell CoS@PPy composites were synthesized using a simple hydrothermal method and *in situ* polymerization. On tuning the filler loading ratio, the complex showed remarkable microwave absorbing performance. In particular, when the doping ratio was 20 wt %, the maximum R_L_ of −41.8 dB for CoS@PPy composites reached 6.96 GHz, along with an absorbing bandwidth (R_L_ < −10 dB) of 5.4 GHz and a matching thickness of 2.05 mm. The impedance matching and improved interfacial polarization might be due to abundant heterointerfaces, and dielectric loss could be considered as the significant reason for the excellent microwave absorbing performance of CoS@PPy composites. Therefore, it is expected that these lightweight and high performance CoS@PPy composites could be key in the fabrication of promising microwave absorbers.
